# Real-time correction of respiratory-induced cardiac motion during electroanatomical mapping procedures

**DOI:** 10.1007/s11517-016-1455-3

**Published:** 2016-03-25

**Authors:** R. van Es, F. J. van Slochteren, S. J. Jansen of Lorkeers, R. Blankena, P. A. Doevendans, S. A. J. Chamuleau

**Affiliations:** 1 Department of Cardiology, University Medical Centre Utrecht, E03.511, P.O. Box 85500, 3508 Utrecht, The Netherlands; 2 Interuniversity Cardiology Institute of the Netherlands (ICIN), Utrecht, The Netherlands; 3 Department of Technical Medicine, Faculty of Science and Technology, University of Twente, Enschede, The Netherlands

**Keywords:** Electroanatomical mapping, Respiratory-induced cardiac motion, CMR, Cardiac imaging

## Abstract

Treatment planning during catheter interventions in the heart is often based on electromechanical tissue characteristics obtained by endocardial surface mapping (ESM). Since studies have shown respiratory-induced cardiac motion of over 5 mm in different directions, respiratory motion may cause ESMs artifacts due to faulty interpolation. Hence, we designed and tested a real-time respiration-correction algorithm for ESM. An experimental phantom was used to design the correction algorithm which was subsequently evaluated in five pigs. A piezo-respiratory belt transducer was used to measure the respiration. The respiratory signal was inserted to the NOGA^®^XP electromechanical mapping system via the ECG leads. The results of the correction were assessed by measuring the displacement of a reference point and the registration error of the ESM on a CMR scan before and after correction. In the phantom experiment, the reference point displacement was 6.5 mm before and 1.1 mm after correction and the registration errors were 2.8 ± 2.2 and 1.9 ± 1.3 mm, respectively. In the animals, the average reference point displacement (apex) was reduced from 2.6 ± 1.0 mm before to 1.2 ± 0.3 mm after correction (*P* < 0.05). The in vivo registration error of the ESM and the CMR scan did not significantly improve. Even though the apical movement appreciated in pigs is small, the correction algorithm shows a decrease in displacement after correction. Application of this algorithm omits the use of the time-consuming respiratory gating during ESM and may lead to less respiratory artifacts in clinical endocardial mapping procedures.

## Introduction

Treatment planning during catheter interventions in the heart is often based on electromechanical tissue characteristics [2, 4] obtained by endocardial surface mapping (ESM). ESM systems determine the exact, real-time 3-dimensional (3D) location of the catheter tip inside the heart using an electrical or magnetic field, induced in the thorax of the patient [2, 16]. Measurements of the local depolarization potentials and relative cardiac wall motion are taken on the endocardium and stored with their respective 3D locations to create a 3D reconstruction of the endocardium. Data are interpolated in the areas that are spatially located between the acquired mapping points to reconstruct a continuous endocardial surface.

During the ESM procedure, the patient is usually awake and breathing freely. Respiratory-induced cardiac motion (RICM) leads to spatial errors in the ESM. A low spatial resolution of ESM together with the RICM-induced spatial artifacts may cause inaccurate positioning of endocardial ablations or intramyocardial injections in the context of cardiac regenerative therapy. Studies in patients using different cardiac imaging modalities report RICM ranging up to a centimeter during free breathing, mainly in the caudal–cranial direction [7–9, 11, 12]. Since a sub-centimeter precision is often required for effective therapy, a technique to overcome interference of RICM during ESM procedures is desirable.

Fusion of ESM data with surface meshes obtained from pre-procedurally acquired 3D imaging data from, e.g., CMR or SPECT/CT has recently gained more interest in order to optimize catheter interventions [3, 5, 10, 11, 13, 14]. Since registration of the imaging modalities is done using landmark or surface registration, minimization of the spatial error is of great importance. RICM artifacts can lead to a misalignment in the registration process; therefore, compensation for RICM without the use of respiratory gating [1] will reduce procedure time and improve registration accuracy.

In this study, we introduce a novel technique to correct for the effect of RICM during ESM procedures without the need for respiratory gating. The NOGA^®^XP (Biosense Webster, Cordis, Johnson & Johnson, version 1.1.43) intramyocardial injection system was used which provides a 3D magnetic tracking of the catheter and measurement of local electrical and mechanical tissue characteristics. Since the NOGA^®^XP system is not able to measure respiration, a simple add-on was developed to enable respiratory registration through available electrocardiogram (ECG) leads.

We identified the effect of RICM on the ESM accuracy during mapping procedures by comparing ESM data with gold standard CMR-derived surface meshes in a porcine study. The goal was twofold: (1) to minimize RICM artifacts in constructed ESM and (2) to decrease the registration error in the fusion of ESMs with CMR.

## Methods

### Respiration and NOGA measurements

**Fig. 1:**
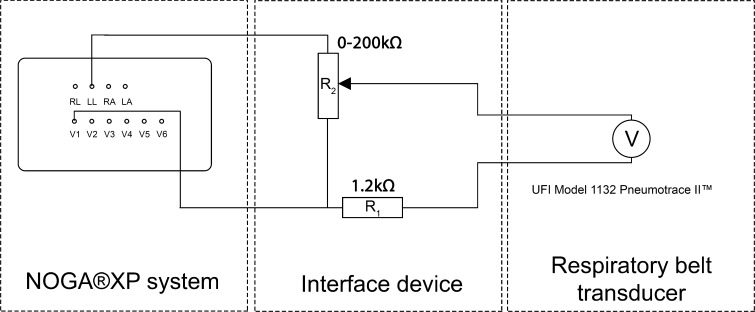
Circuit of the system used to connect a respiratory belt transducer to the ECG leads of NOGA^®^XP system to instigate ECG baseline drift in the V1 ECG channel. The interface device used to alter the voltage, delivered by the respiratory belt transducer, is shown in the *center panel*. The interface device is connected to the left leg (LL) and V1 ECG channels on the NOGA^®^XP PUI box. The respiratory belt transducer is connected to the interface device using a regular BNC connector

A piezo-respiratory belt transducer (UFI Model 1132 Pneumotrace II™) was connected to an ECG input of the NOGA^®^XP system (Fig. 1). The respiratory signal was represented on the NOGA^®^XP system as a baseline shift of the connected ECG channel. Prior to the experiment, the potentiometer in the device was adjusted so that the connected ECG channel showed a clear baseline drift. The NOGA^®^XP system stores end-diastolic 3D location of each acquired location in the local database using the built-in ECG *R*-wave triggering. The NOGA^®^XP system records a 12-lead ECG with a length of 2.5 s per measured point. The sample rate of the signal is 1 kHz. The NOGA^®^XP ECG filter settings were optimized to allow visualization of respiratory signals in the 0.05–0.5 Hz range. An external computer was used to access the ECG data on the NOGA^®^XP system via a shared folder. The NOGA^®^XP database was accessed through a SQL server with read-only permissions.

### Phantom experiment

A non-rotational symmetric bottle was mapped from the inside in a stationary position. Thereafter, the phantom was mounted on a test lung that was connected to a ventilator. The ventilator tidal volume was set to induce a 15-mm motion of the phantom in primarily one direction. To acquire a reference measurement, the 7 French NOGA^®^XP mapping catheter (Biosense Webster, Cordis, Johnson & Johnson, Diamond Bar, USA) was fixed to the phantom while ventilating the test lung. Hereafter, the phantom was mapped on the inside while ventilating the test lung. During the mapping procedures, an ECG simulator was connected to the NOGA^®^XP system to enable triggered data acquisition. The ECG simulator was set to simulate normal sinus rhythm at 80 beats/min.

### Porcine experiments

All animal experiments were conducted in accordance with the national guidelines on animal care and with prior approval by the Animal Experimentation Committee of the Faculty of Medicine, Utrecht University, the Netherlands. Five female Dallas Landrace pigs were subjected to a 90-min left anterior descending balloon occlusion and subsequent reperfusion 8 weeks before the measurements.

A respiratory belt transducer was attached around the abdomen in the sub-thoracic region, where maximal respiratory-induced abdominal movement could be measured. The transducer was connected to the ESM system as described before. The ventilator was set to a frequency of 12 breaths per minute with a tidal volume of 800 ml and a 2:1 expiration to inspiration ratio. The NOGA^®^XP catheter was inserted into the left ventricle (LV) via the left carotid artery and retrograde passage through the aortic valve. First, the catheter was positioned in the LV apex and a reference measurement was taken containing at least 60 measurements to assure measurements in all phases of the respiratory cycle. Secondly, in an independent procedure, a complete map of the LV was made. After the mapping procedures, a CMR scan was made. The animals were euthanized after finishing the experiments.

### CMR

In vivo CMR was performed under anesthesia on a clinical 3T scanner (Achieva TX, Philips Healthcare, Best, the Netherlands) with a 32-channel receiver coil.

ECG-gated cine images were made with voxel size = 2 × 2 mm, flip angle = 45°, slice thickness = 8 mm, field of view = 320 × 320 mm, repetition time [TR] = 3.2 ms, echo time [TE] = 1.6 ms and 30 phases/R-to-R interval.

End-diastolic frames of the cine images were segmented using the freely available software Segment version 1.9 R3262 (http://segment.heiberg.se) [6]. An end-diastolic endocardial surface mesh was generated and used for the registration of the NOGA^®^XP mapping points. The mesh creation and registration were performed using the in-house developed 3D CartBox and were based on a standard rotation and translation followed by an angle-limited iterative closest point procedure and manually corrected if necessary [14].

### Correction algorithm

**Fig. 2:**
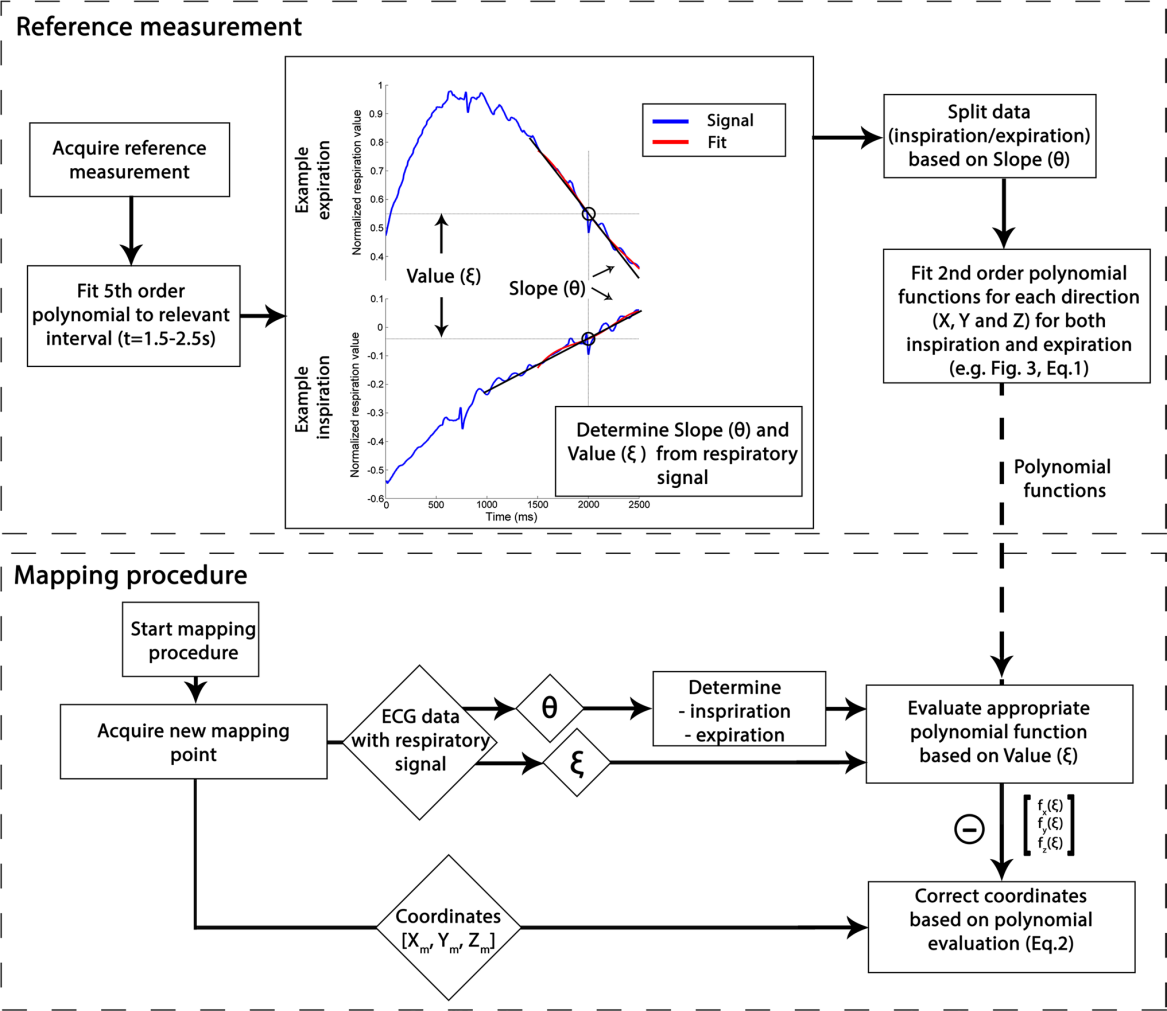
The *upper panel* shows the flowchart of the algorithm that was used to determine the correction parameters from the reference measurement. The *bottom* how these parameters were used to correct acquired point during the mapping procedure. *θ* = slope, *ξ* = *y* value, *X*_m_ = measured coordinate

**Fig. 3:**
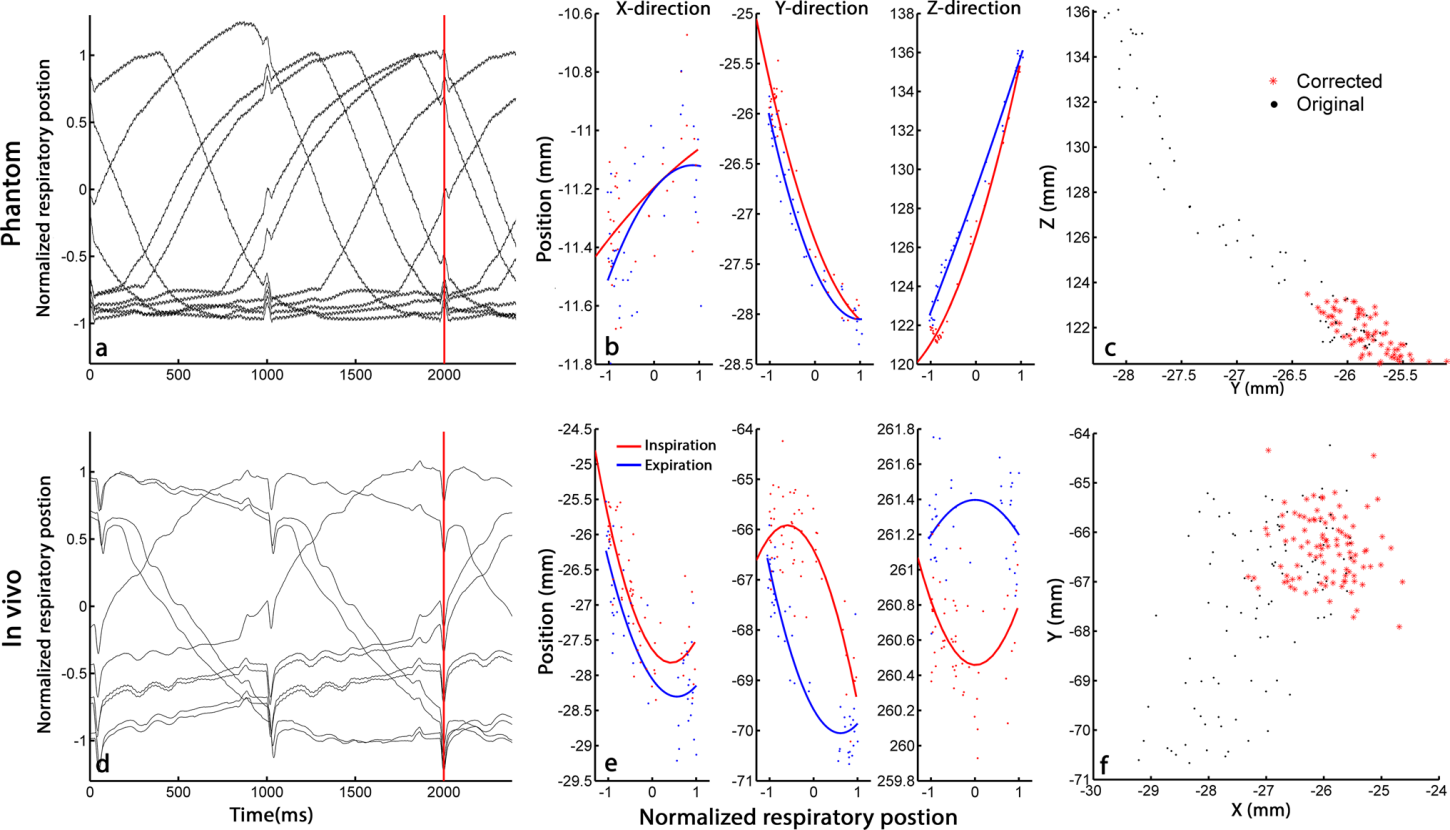
**a**, **d** Example of the first ten registered respiratory signals recorded on the precordial ECG V1 lead in the phantom and in vivo experiments, respectively. The respiratory position is determined at time point *t* = 2 s (*red line*), the end-diastolic phase (*R*-wave). **b**, **e** The recorded 3-dimensional (*X*, *Y* and *Z*) data of all (*n* = 77 and *n* = 100, respectively) mapped reference points with the fitted second-order polynomial functions for inspiration (*red*) and expiration (*blue*). Note the differences in scale on the *vertical* axis. **c**, **f** Reference measurement before (*black*) and after correction (*red*). This is a 2-dimensional representation of the 3D data in the two *directions* that show the largest variation

A detailed overview of the correction algorithm is shown in Fig. 2. As a first step, a reference measurement was taken to assess the RICM. In the phantom experiment, the catheter tip was mounted at a fixed surface location during movement of the phantom. In the in vivo experiments, the catheter was positioned in the LV apex during the reference measurements. In both experiments, at least 60 measurements were used for the reference measurement. It was assured that points and measurements were located in every phase of the respiratory cycle. Storage of the 3D location of the catheter tip is triggered at *t* = 2 s in the stored ECG. For the respiratory analysis, the ECG signal containing the respiratory data was fitted with a fifth-order polynomial function over the 1.5- to 2.5-s interval to eliminate *R*-wave artifacts and signal noise (MATLAB 2013a version 8.1, The MathWorks Inc.). The data were split into an inspiratory and expiratory part based on the slope (*θ*) of the respiratory signal at *t* = 2 s (Fig. 2a). For both parts, the position is defined in the *X*, *Y* and *Z* directions. For each direction, a second-order polynomial function is fitted using a least squares approach (Eq. 1) (e.g., Fig. 3b, e). The six resulting functions were thereafter used during the mapping procedure to calculate the correction of every acquired point based on the value (*ξ*) and *θ* of the respiratory signal (Eq. 2).

1 $$ f_{c} = ax^{2} + bx + c $$

The second-order polynomial function that was used to fit the data acquired during the reference measurement. This was done for each direction for both the inspiratory and expiratory data. *f*_c_ represents the measured values, and parameters *a, b* and *c* are determined and used to subsequently calculate the correction during the mapping procedure.

2 $$ \left[ {\begin{array}{*{20}c} {x_{\text{c}} } \\ {y_{\text{c}} } \\ {z_{\text{c}} } \\ \end{array} } \right] = \left[ {\begin{array}{*{20}c} {x_{\text{m}} } \\ {y_{\text{m}} } \\ {z_{\text{m}} } \\ \end{array} } \right] - \left[ {\begin{array}{*{20}c} {f_{\text{x}} \left( \xi \right)} \\ {f_{\text{y}} \left( \xi \right)} \\ {f_{\text{z}} \left( \xi \right)} \\ \end{array} } \right] $$

The equation that was used to correct the acquired mapping data, *f*_*x*_ *(ξ),* represents the polynomial function best fitting the *X*-coordinate data from the reference measurement shown in Eq. 1, and *ξ* represents the *y* value measured in the normalized respiratory signal. The corrected and measured *X*-coordinate is represented by *X*_c_ and *X*_m_, respectively.

### Analysis and statistics

The algorithm is used to relocate measurement points to their location at maximal expiration. To assess the effects of the RICM correction, the root-mean-squared error (RMSE) of the geometrical distance from all points of the reference measurement to the point acquired at maximal expiration was calculated. The same end-expiratory location was used for both the uncorrected and corrected RMSE. Maximal apical displacement represents the maximal range of all apical locations during the reference measurement, i.e., the distance between the point at maximal expiration and the point furthest from that. Both RMSE and the maximal apical displacement are expressed in millimeters. The registration error is calculated as mean distance of each mapping point to the CMR end-diastolic endocardial surface mesh after registration of the NOGA^®^XP ESM with the endocardial surface mesh generated from cine CMR. The registration error is expressed as mean ± standard deviation in millimeters. Paired *t* tests were used to compare original with corrected data, and *p* < 0.05 was considered to be statistically significant.

## Results

### Respiratory registration

Recording respiratory signals on a precordial ECG lead were performed successfully. The respiratory-induced baseline shift in ECG lead V1 during the phantom and in vivo experiments is shown in Fig. 3a, d, respectively.

### Phantom experiment

**Fig. 4:**
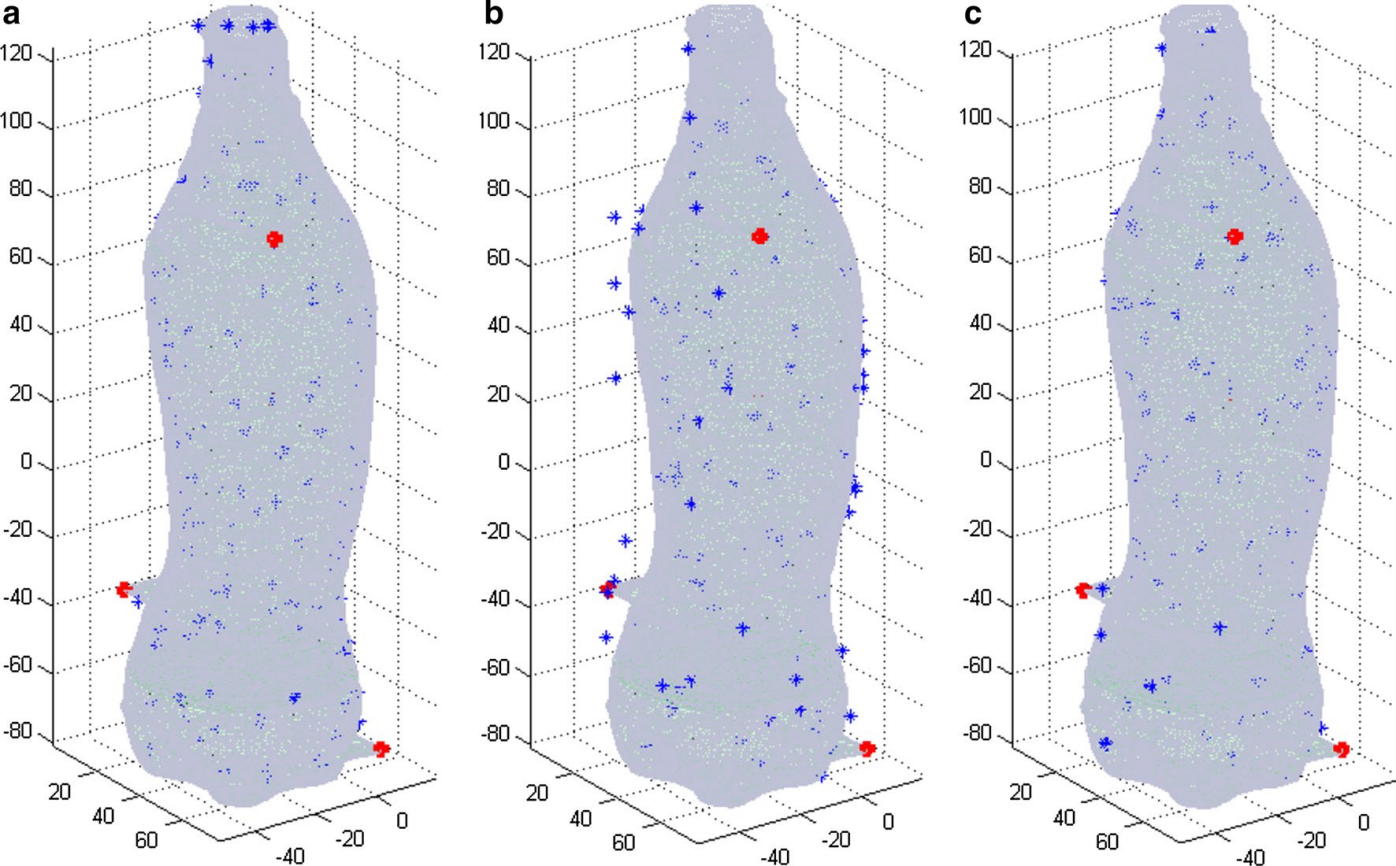
**a** The registration of acquired ESM points (*blue*) to the MRI-derived surface mesh (*light blue*) where no motion was applied to the phantom. Landmark points (*red*) were fixed to the external surface of the phantom (registration error 2.0 ± 1.4 mm). **b** The uncorrected registration with applied motion (registration error 2.8 ± 2.2 mm). **c** The corrected registration with applied motion (registration error 1.9 ± 1.3 mm)

**Fig. 5:**
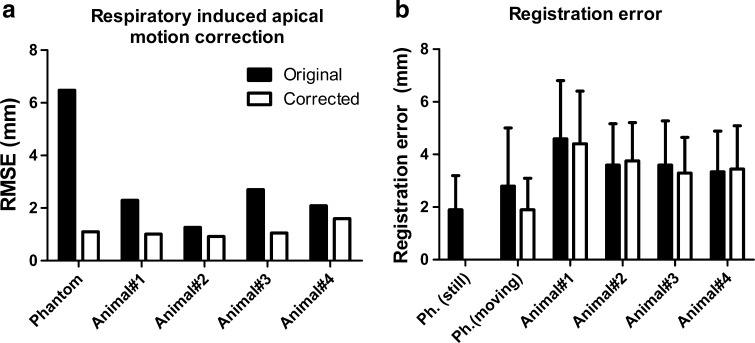
**a** The RMSE error of the reference measurement before and after correction. **b** The registration error of the ESM map and the MRI-derived endocardial surface mesh before and after correction. The phantom where no motion was applied (Ph. still) was not corrected. *Ph.* phantom

In the motionless phantom, 142 positions were mapped; in the moving phantom, 70 and 118 points were, respectively, acquired during the reference and mapping measurements. In the reference measurement, the derived motion of the phantom was 15 mm in the *Z* direction and 2 mm in the *X* and *Y* directions (Fig. 3b). The result of the correction algorithm on the motion of the reference measurement is shown in Fig. 3c. The registration error of the ESM on the CMR was 2.0 ± 1.4 mm when no respiratory motion was applied to the phantom (Fig. 4a). With motion, the registration error was 2.8 ± 2.2 mm before and 1.9 ± 1.3 mm after correcting for the motion (Figs. 4b, c and 5b; Table 1). The RMSE in the reference measurement was 6.5 mm before and 1.1 mm after correction (Fig. 5a).

**Table 1:** Individual results of all experiments

	Apex movement RMSE (mm)		Apex maximal displacement (mm)		Registration error (mm)	
	Original	Corrected	Original	Corrected	Original	Corrected
Phantom	6.5	1.1	14.0	2.02	2.8 ± 2.2	1.9 ± 1.2
Animal#1	2.3	1.0	3.4	1.95	4.6 ± 2.2	4.4 ± 2.0
Animal#2	1.3	0.9	2.6	1.67	3.6 ± 1.6	3.8 ± 1.5
Animal#3	2.7	1.6	5.5	2.62	3.6 ± 1.7	3.3 ± 1.4
Animal#4	4.1	1.7	3.3	3.06	3.3 ± 1.6	3.4 ± 1.7

### Porcine experiments

In five animals (83 ± 6 kg), on average, 91 (range 75–101) reference points were acquired in the apex and the mean number of points in the left ventricular maps was 80 (range 63–90). In four animals, the baseline shift in the respiratory signal was successfully recorded. The RICM correction algorithm was able to correct for the respiratory-induced apical movement (Fig. 5a; Table 1). The average apical displacement (RMSE) was reduced by 51 % from 2.6 ± 1.0 mm before to 1.2 ± 0.3 mm after correction (*p* < 0.05). The maximal apical displacement was reduced by 34 % from 3.7 ± 1.1 mm before to 2.3 ± 0.5 mm after correction (*p* = 0.09). A representative example of acquired apical reference points is shown in Fig. 3f. The registration error was not significantly (*p* = 0.62) reduced from 3.8 ± 0.5 to 3.7 ± 0.4 mm (mean ± SEM) after correction.

## Discussion

Measuring RICM with the NOGA^®^XP system is feasible using our in-house developed customized interface. In a phantom experiment, the correction algorithm showed to be effective in reducing the RMSE of the reference measurement and the registration error of the NOGA^®^XP ESM and the CMR dataset. In the porcine experiments, RICM of the LV apex was smaller than values reported in human studies. Our correction algorithm is, however, capable of significantly reducing (34 %) the apical displacement throughout the respiratory cycle. The reduction in registration error of corrected ESM data on the CMR data was minimal. This most likely is due to the minimal amount of RICM observed in this porcine model. One animal was excluded from analysis due to a malfunctioning respiration transducer.

Multiple studies into RICM report movement of the human heart in the range of one centimeter [7–9, 11, 12]. In the present study, the maximal apical displacement measured was 3.7 ± 1.1 mm. An explanation for this difference might be found in the difference of thoracic anatomy between humans and pigs. The thoracic anatomy of the pig might lead to reduced RICM. Furthermore, to explore the RICM of pigs, we set up a single experiment in which we increased the tidal volume. We observed a 120 % increase (3.3–7.3 mm) in RICM (maximal apical displacement) of the apex upon a 20 % increase (800–950 ml) in tidal volume. Although a tidal volume of 800 ml was used during the experiments, the design and validation of the RICM algorithm can be done accurately with the used setup.

Multiple commercially available ESM systems offer the option to use respiratory gating in addition to ECG gating during ESM procedures. This inevitably leads to longer procedure times since mapping points are only acquired during a short phase of the respiratory cycle, usually the end-expiratory phase [1]. Furthermore, respiratory gating in free-breathing patients may be unreliable since respiratory cycles are never identical. This leads to different residual volumes and thus to different positions of the heart during end expiration. Our respiratory correction algorithm allows the acquisition of mapping points during the whole respiratory cycle. By uncoupling inspiration and expiration, and taking both the slope (*θ*) and value (*ξ*) into account during the analysis, the algorithm is able to minimize problems associated with breath to breath variability in tidal volume. Since the algorithm depends on reference measurements, subject-specific breathing characteristics, such as depth and rate of breathing, are considered in the algorithm. Additionally, using these characteristics, abnormal breaths during the mapping procedure can be identified automatically and excluded if necessary.

### Data acquisition

Acquiring respiration data with the NOGA^®^XP system using ECG input ports are feasible since our customized device uses only one of the precordial leads (e.g., V1), which are often unused during mapping/injection procedures. The use of an ECG lead as an input for the respiratory signal ensures synchronicity of the stored signals and location data. For use in a clinical setting, a dedicated solution is desirable to allow different settings of, for example, the filter settings. In this study, no difficulties in visualizing neither the ECG signals nor the respiratory signals were found.

### Limitations

All experiments were performed with the animal connected to a ventilator; therefore, respiration curves were predictable and similar throughout the entire procedure. RICM in the porcine model is minimal, and as a result, it is difficult to predict the effect of RICM on the registration error in a clinical situation.

### Clinical perspective

Many imaging modalities, such as late gadolinium enhancement CMR, provide detailed information about the infarct location and its characteristics. Combining or fusing ESM data with, for example, late gadolinium enhancement CMR may improve therapeutic outcome [10, 13, 16]. The registration error between ESMs and, for example, MRI data is typically around 3 mm in animals and humans (e.g., 3.22 ± 1.86 mm [14] and 3.8 ± 0.6 mm [15], respectively). Since RICM in patients is often much larger [7–9, 11, 12], correction of RICM will probably lead to fewer registration artifacts and therefore to more accurate multimodality catheter diagnosis and treatment. The validation of the correction algorithm in this study was performed retrospectively. However, the correction algorithm is intended to be used in real time during ESM mapping procedures. For example, the reference measurement takes about 1–2 min, and calculating parameters needed for the subsequent procedure requires less than 2 s on a modern computer. During the actual mapping procedure, calculating the correction for a single acquired point requires a few milliseconds. Use of the correction algorithm in real time will therefore not affect the length of the ESM procedure.

## Conclusion

The correction of RICM during ESM procedures by our correction algorithm is feasible and can be incorporated in commercially available systems. In contrast to the phantom experiments, the suggested RICM correction method was of less additional value during porcine experiments due to minimal RICM in the porcine model. Nevertheless, during patient procedures, RICM correction can be of additional value to obtain an anatomically correct map and allows a more optimal registration with respiratory-gated cardiac imaging data. A new clinical study must be performed to investigate the clinical benefit of RICM correction.
